# The Dual Impact of Time and Content Exposure of Social Media on Diabetes Self-Management in Older Adults: Cross-Sectional Study

**DOI:** 10.2196/67312

**Published:** 2025-09-18

**Authors:** Qingyuan Ye, Ruiyang Xu, Li Li, Meng Zhao, Shan Wang, Sijing Peng, Si Chen, Fatema Ahmed, Chen Wu, Kefang Wang

**Affiliations:** 1 School of Nursing and Rehabilitation Shandong University Jinan China; 2 School of Nursing Anhui University of Chinese Medicine Hefei China

**Keywords:** social media, media exposure, type 2 diabetes mellitus, self-management, older adults

## Abstract

**Background:**

Self-management is critical for older adults with type 2 diabetes mellitus (T2DM); however, its practice remains suboptimal. Social media has become an accessible and effective stimulus source for the public, which has the potential to promote health behaviors, but its effect on the self-management of older adults with T2DM remains unknown.

**Objective:**

We aimed to investigate the relationship between social media exposure, specifically time exposure and content exposure, and the self-management of older adults with T2DM.

**Methods:**

In this cross-sectional study, we enrolled 257 older adults with T2DM who used short-form video apps from community health care centers. We assessed subjective and objective time and content exposure. We transformed text-based content exposure into diabetes-related content exposure encompassing irrelevant, harmful, hypobeneficial, and hyperbeneficial categories using Q-methodology. Self-management was assessed through a validated questionnaire. We used restricted cubic splines and linear regression models to model the relationships between time exposure and content exposure and self-management, respectively.

**Results:**

Of 257 older adults with T2DM, the median age was 69 (IQR 65-72) years, 53.3% (n=137) were women, the mean sum score of self-management was 35.7 (SD 10.4), the median subjective time exposure was 120 (IQR 60-120) minutes, and 61.1% (n=157) of them were exposed to hyperbeneficial content. There was an approximate L-shaped dextrorotatory relationship between time exposure and self-management, with a decline in self-management when time exposure surpassed 139.8 minutes daily. Exposure to hyperbeneficial content was positively associated with the overall self-management (B=3.46, 95% CI 0.71-6.21). For participants exposed for more than 139.8 minutes daily, this positive association remained robust (B=7.27, 95% CI 1.54-13.00). In subdimensional analyses, hyperbeneficial content exposure was positively associated with general diet (B=1.51, 95% CI 0.54-2.49) and blood glucose testing (B=1.31, 95% CI 0.25-2.38).

**Conclusions:**

Social media exposure presented a double-edged sword for self-management of older adults with T2DM. Self-management declined when the daily time spent on social media exceeded 139.8 minutes. However, exposure to hyperbeneficial content was associated with better self-management of individuals, regardless of excessive time spent on social media. Future longitudinal and experimental studies that validate the multifaceted association between social media exposure and health behaviors are needed. If confirmed, these findings would support the implementation of media prescription programs by health care providers in communities.

## Introduction

### Background

Type 2 diabetes mellitus (T2DM) imposes a substantial burden on health care systems worldwide [1], with older adults comprising over half of the adults diagnosed with T2DM worldwide [2]. Satisfactory self-management is crucial for older adults with T2DM, without which individuals are more likely to experience diabetes-associated complications, functional disability, and premature mortality [3]. Self-management is influenced by output information from external stimuli [4]. The lifestyle interventions are taken as the focal stimulus and function through providing reliable knowledge, regulated guidance, and interpersonal support theoretically [5], but face challenges of participants’ low compliance and lack of perseverance in practice [6,7]. Therefore, the search for other optional and effective stimulus sources among older adults with T2DM is warranted.

Social media, which is abundant in disease-related information, can serve as a stimulus source for older adults with T2DM who frequently use these platforms to seek information about their condition [8]. Efforts from the government and businesses to bridge the digital divide have caused social media to explode in popularity among the older population. In the United States, 45% of individuals aged >65 years were active users of social media [9], while in China, older users make up 11.7% of all short-form video app users [10]. Instead of using text- or image-based platforms such as Twitter, most older adults prefer short-form video apps [11]. Social media is a powerful medium to promote health behaviors [12]. Supported by the cultivation theory [13] and empirical evidence, individuals’ behavior may be influenced by the content they are exposed to on social media. Research demonstrated that exposure to COVID-19–related information on social media was associated with individuals’ better practice of hand sanitation [14]. Ngqangashe and Backer [15] found that exposure to culinary videos on social media was associated with the food choice behaviors of middle school children. In addition, as an important dimension of social media exposure, time exposure on social media would also impact individuals’ health behaviors. Zhang et al [16] found that women who reported excessive media use (>3 h/d) were more likely to have unhealthy lifestyles, including reduced physical activity, inadequate dietary diversity, and poorer sleep quality. Wu et al [17] found that longer-time exposure to short-form video was associated with decreased steps and time spent in physical activity in older women. However, multiple pitfalls in the literature have compromised the soundness of the existing findings. First, previous studies have reported significant but inconsistent associations between time and content exposure and health behaviors, highlighting that social media exposure has distinct characteristics that differentially influence self-management. Therefore, it is essential to examine both characteristics concurrently. Second, research often assumes a linear relationship between time exposure and the outcome, which has not been empirically confirmed. Third, the content that individuals are exposed to on social media is abundant, whereas researchers often collect specific content through yes or no questions. Such a practice fails to capture the full range of content that individuals are exposed to on social media [18]. Fourth, content exposure and time exposure have often been evaluated in studies with self-reported questions without considering the potential recall bias. Finally, the potential of social media to elicit self-management of older adults with T2DM has not been clarified.

### Objectives

This study aimed to explore the role of social media as a source of stimuli in promoting self-management among older adults with T2DM and to investigate the associations between social media exposure (both content exposure and time exposure) on short-form video platforms and self-management of older adults with T2DM using both subjective and objective measures.

## Methods

### Ethical Considerations

The ethical oversight of this cross-sectional study was provided by the Shandong University (2023-R-017). All eligible adults provided permission to access their short-form video app use records, including time spent on each app, following, liking, bookmarking, and browsing history; agreed to provide deidentified individual information for research; and signed a written consent form before participating. This study followed the Strengthening the Reporting of Observational Studies in Epidemiology (STROBE) reporting guidelines. A standardized monetary incentive (gift) was provided to encourage participation.

### Recruitment

From February to June 2023, we launched a cross-sectional study named Media on Diabetes. A convenience sampling approach was used in this study, and upon the director’s approval of the 5 community health centers, data were collected at the community health centers in Jinan, Shandong Province, China. Older people with T2DM were recruited during their routine physical examination visits. The inclusion criteria of the Media on Diabetes study were set as being aged ≥60 years, having been diagnosed with T2DM for at least 1 year, and using the short-form video apps Douyin or TikTok, Kuaishou, Haokan, and Toutiao on smartphones. Adults were excluded if they had been diagnosed with acute diabetes complications or physical disorders that could additionally affect their self-management, including cerebral ischemia, New York Health Association classes III and IV, otoliths, gout, severe asthma, chronic kidney disease at stage III or above, chronic obstructive pulmonary disease (Global Initiative for Chronic Obstructive Lung Disease III and IV), or moderate or severe swallowing disorders. We also excluded adults who were unable to walk independently even with walking aids, diagnosed with mental or cognitive disorders, diagnosed with hearing impairment and not wearing a hearing aid, or without diagnosed hearing impairment but failing to hear when called aloud. The principal investigator and 2 trained research assistants collected data in the field. Adults filled in the questionnaires by themselves, and if they had inadequate reading capacity, the investigators assisted in reading questions. A total of 268 participants were recruited for this study, among whom 11 (4.1%) declined to complete the survey. Consequently, 257 valid questionnaires were included, yielding a response rate of 95.9%.

### Sample Size

We used the formula 
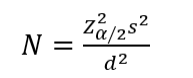
 to calculate the sample size for this study [19]. The formula is used for cross-sectional studies, where Z_α/2_ denotes the critical value for a 2-sided test at a 95% CI, *s* represents the SD derived from a previous study, and *d* represents the desired margin of error for estimating the population mean [19]. The mean (SD) of the sum score of self-management in the previous study was 37.5 (14.0) [20]; therefore, we took the values of Z_α/2_=1.96, *s*=14.0, and *d*=mean×5%=1.875. By adding 10% of adults to account for any withdrawal (eg, time conflicts, loss of interest, or privacy concerns), the minimal sample size for this study was calculated to be 235.

### Measures

#### Social Media Exposure Assessment

Variables on the use of social media included social media exposure duration, exposure to multiplatforms, the feature of exposure, subjective time exposure, objective time exposure, and diabetes-related content exposure. Subjective time exposure was assessed through a self-reported question, and objective time exposure was recorded using data from the short-form video apps to account for potential recall bias. Participants were asked to indicate the types of content they watched from a list of 47 options (eg, health care, current affairs, and sport events) derived from a previous investigation on older adults [17], with space for additional comments. In addition, participants’ interactions, such as liking, bookmarking, and browsing history on the short-form video apps, were gathered to supplement their self-reported data. Details of these variables are depicted in Table S1 in Multimedia Appendix 1 [21-24].

#### Self-Management Assessment

Diabetes self-management was assessed with the Chinese version of the Summary of Diabetes Self-care Activities, which was well adapted to Chinese culture [25]. It assessed 6 domains of self-management, including general diet, specific diet, exercise, blood glucose testing, foot care, and medication use of adults with T2DM. Each item was graded from 0 to 7, except for the item of high-fat food eating within the specific diet dimension, which was scored in reverse. The sum score of the Summary of Diabetes Self-care Activities ranged from 0 to 77, with higher scores demonstrating better self-management.

#### Covariates Assessment

By reviewing the literature on factors influencing diabetes self-management [26,27], covariates considered in the analyses included social demographic variables of sex, age, educational background, living status, marital status, working status, and monthly household income per capita; health-related variables of BMI, pain, diabetes duration, medication related to diabetes, complications of diabetes, multimorbidity, smoking history, and sleep duration; and psychological variables of self-efficacy, perceived stress, and social support. On the basis of the proposition of cultivation theory, which posits that traditional media exposure shapes health behaviors [13], we also considered variables of daily smartphone use time and traditional media use variables of self-reported traditional media exposure and health-related content exposure on traditional media. Coding and grading of the variables are shown in Tables S2 and S3 in Multimedia Appendix 1.

### Statistical Analysis

The mean imputation approach was used to handle the missing data issue for the continuous variables of social media exposure duration (0.8%), self-efficacy (1.6%), perceived stress (1.2%), and social support (1.2%). Q-methodology is a robust approach that converts qualitative data into quantitative form and is widely applied in empirical studies [28,29]. In this study, we used Q-methodology [30] to assign scores to each social media content based on its relevance to T2DM and categorized participants according to their exposure to diabetes-related content. The procedures are outlined in Figure S1 in Multimedia Appendix 1. Participants who were exposed to irrelevant content only were coded as irrelevant content exposure, those exposed to harmful content with or without irrelevant content were coded as harmful content exposure, those exposed to beneficial content with a relevant score between 1 and 3 were coded as hypobeneficial content exposure, and those exposed to beneficial content with a relevant score of 4 or 5 were coded as hyperbeneficial content exposure.

The Kolmogorov-Smirnov goodness-of-fit test was used to assess the normality of continuous variables, which were described as mean and SD or median (IQR), as appropriate. Categorical variables were described as frequencies and percentages. The sum score of self-management was normally distributed, and as appropriate, the associations were calculated with Pearson or Spearman correlation for continuous variables and 2-sample *t* tests or ANOVA for categorical variables. Scores of self-management dimensions were not normally distributed, and we then used Spearman correlation, the Mann-Whitney *U* test, and the Kruskal-Wallis H test to test their associations with continuous and categorical variables. Consequently, covariates of the sum score of self-management and each score of self-management dimensions were selected a priori.

We used Spearman correlation to test the association between subjective time exposure and objective time exposure, and the correlation coefficient was 0.91 (*P*<.001), indicating that the recall bias was marginal. Therefore, we used subjective time exposure instead of objective time exposure for subsequent statistical inference due to its greater practical feasibility in community settings and comparability with the existing literature.

A scatter plot diagram was used to depict the relationship between time exposure and the sum score of self-management, and a nonlinear relationship was found. Therefore, the nonlinear relationship was estimated using a restricted cubic spline with the estimator of ordinary least squares [31], while controlling for significant covariates. We repeated the steps to test the relationships between time exposure and each score of the self-management dimensions. A *P* value for an overall <.05 indicates the relationship between time exposure and self-management is statistically significant, and a *P* value for a nonlinear <.05 indicates a significant nonlinear relationship.

Multiple linear regression models were used to test the relationships between diabetes-related content exposure and the sum score of self-management; generalized linear models were used to evaluate the correlations between diabetes-related content exposure and scores of the self-management dimensions. If the nonlinear relationships between time exposure and self-management scores were found, we used the same modeling strategies to evaluate the associations between diabetes-related content exposure and self-management scores in subgroups based on time exposure, as appropriate.

All the analyses were performed with R (version 4.3.1; R Program for Statistical Computing), and 95% CIs for model estimates were reported. A 2-sided *P*<.05 was considered statistically significant. For diabetes-related content exposure as an independent variable, Bonferroni adjustment was applied due to multiple comparisons, and the threshold for statistical significance was adjusted to *P*<.017 (0.05/3=.017). Data were analyzed from July 2023 to May 2024.

## Results

### Participant Characteristics

Of the 257 participants, 137 (53.3%) were women, the median age was 69 (IQR 65-72) years, and the median diabetes duration was 7 (IQR 2-13) years (Table 1). The mean sum score of self-management of participants was 35.7 (SD 10.4). The median subjective time exposure of the participants was 120 (IQR 60-180) minutes. per day, the median social media exposure duration was 3 (IQR 1-5) years, and 173 (67.3%) were exposed to multiplatforms (Table 2).

**Table 1:** Participants’ characteristics and their associations with self-management (N=257).

Characteristics				Participants		Statistics		*P* value^a^
**Social demographic variables**								
	**Sex, n (%)**					1.239 (*df*=1)^b^		.22
		Male	120 (46.7)					
		Female	137 (53.3)					
	Age (y), median (IQR)			69 (65-72)		–0.045^c^		.47
	**Educational background, n (%)**					1.208 (*df*=3)^d^		.31
		Elementary school or below	37 (14.4)					
		Middle school	79 (30.7)					
		High school	95 (37.0)					
		College or above	46 (17.9)					
	**Living status, n (%)**					0.397 (*df*=1)^b^		.69
		Living alone	21 (8.2)					
		Living with family, relatives, or friends	236 (91.8)					
	**Marital status, n (%)**					–0.435 (*df*=1)^b^		.66
		Married	232 (90.3)					
		Unmarried, divorced, or widowed	25 (9.7)					
	**Working status, n (%)**					–2.705 (*df*=1)^b^		.007^e^
		Retired	234 (91.1)					
		Still working	23 (8.9)					
	**Monthly household income per capita (CNY)^f^, n (%)**					2.473 (*df*=1)^d^		.09
		<2000	30 (11.7)					
		2000-4000	118 (45.9)					
		>4000	109 (42.4)					
**Health-related variables**								
	**BMI (kg/m^2^), n (%)**					2.469 (*df*=2)^d^		.09
		<24.0	103 (40.1)					
		24.0-28.0	109 (42.4)					
		≥28.0	45 (17.5)					
	**Pain, n (%)**					2.939 (*df*=1)^b^		.004^e^
		No	129 (50.2)					
		Yes	128 (49.8)					
	Diabetes duration (y), median (IQR)			7 (2-13)		0.258^c^		<.001^e^
	**Medication related to diabetes, n (%)**					22.693 (*df*=2)^d^		<.001^e^
		None	56 (21.8)					
		Oral medicine	162 (63.0)					
		Insulin	39 (15.1)					
	**Complications of diabetes, n (%)**					0.158 (*df*=1)^b^		.88
		No	187 (72.8)					
		Yes	70 (27.2)					
	**Multimorbidity, n (%)**					–0.017 (*df*=1)^b^		.99
		No	27 (10.5)					
		Yes	230 (89.5)					
	**Smoking history, n (%)**					2.043 (*df*=2)^d^		.13
		Be smoking	31 (12.1)					
		Quit smoking	19 (7.4)					
		Nonsmoker	207 (80.5)					
	Sleep duration, median (IQR), h			6 (6-7)		0.024^c^		.70
**Psychological variables**								
	Self-efficacy, median (IQR)			29 (25-34)		0.249^c^		<.001^e^
	Perceived stress, median (IQR)			15 (9-20.5)		–0.157^c^		.01^e^
	Social support, mean (SD)			39.54 (7.16)		0.138^g^		.03^e^

^a^*P* values from univariate analyses for the sum score of self-management.

^b^Two-sample *t* tests were performed for binary categorical variables, including sex, living status, marital status, working status, pain, complications of diabetes, and multimorbidity.

^c^Spearman correlations were performed for continuous variables that do not conform to a normal distribution, including age, diabetes duration, sleep duration, self-efficacy, and perceived stress.

^d^ANOVA was performed for multicategorical variables, including educational background, monthly household income per capita, BMI, medication related to diabetes, and smoking history.

^e^Statistically significant (*P*<.05).

^f^1 CNY=US $0.14.

^g^Pearson correlation was performed for social support (a continuous variable that conforms to a normal distribution).

**Table 2:** Characteristics of media exposure and their associations with self-management in 257 older adults with type 2 diabetes mellitus.

Characteristics					Participants			Statistics			*P* value^a^
**Traditional media**											
	**Traditional media exposure, n (%)**							0.980 (*df*=1)^b^			.33
		No	75 (29.2)								
		Yes	182 (70.8)								
	**Health-related content exposure on traditional media, n (%)**							–1.210 (*df*=1)^b^			.23
		No	220 (85.6)								
		Yes	37 (14.4)								
	Daily smartphone use (min), median (IQR)			120 (60-240)			0.041^c^			.57	
**Social media**											
	Social media exposure duration (y), median (IQR)			3 (1-5)			0.174^c^			.005^d^	
	**Exposure to multiplatforms, n (%)**							–0.135 (*df*=1)^b^			.89
		No	84 (32.7)								
		Yes	173 (67.3)								
	**The feature of exposure, n (%)**							–0.906 (*df*=1)^b^			.37
		Video-based	113 (44.0)								
		Video- and text-based	144 (56.0)								

^a^*P* values from univariate analyses for the sum score of self-management.

^b^Two-sample *t* tests were performed for binary categorical variables, including traditional media exposure, health-related content exposure on traditional media, exposure to multiplatforms, and the feature of exposure.

^c^Spearman correlations were performed for continuous variables that do not conform to a normal distribution, including daily smartphone time use and social media exposure duration.

^d^Statistically significant (*P*<.05).

Of the diabetes-related content exposure variable, hyperbeneficial content comprises 5 core diabetes self-management components: dietary control, physical activity, medication adherence, blood glucose monitoring, and foot care. In contrast, hypobeneficial content includes information about diabetes-related comorbidities, for example, hypertension, dyslipidemia, and coronary heart disease. While clinically relevant, such content offers generalized health benefits rather than diabetes-specific advantages. The distribution of participants across the different categories of diabetes-related content exposure is presented in Table S4 in Multimedia Appendix 1. There were 64 (24.9%) participants who fell into the category of irrelevant content exposure, 1 (0.4%) who fell into the category of harmful content exposure, 35 (13.6%) who fell into the category of hypobeneficial content exposure, and 157 (61.1%) who fell into the category of hyperbeneficial content exposure.

For the univariate analyses presented in Table 1, significant associations with the sum score of self-management were identified for working status (*t_1_*=–2.705; *P*=.007), pain (*t_1_*=2.939; *P*=.004), diabetes duration (*r*=0.258, *P*<.001), medication related to diabetes (*F_2_*=22.693, *P*<.001), self-efficacy (*r*=0.249, *P*<.001), perceived stress (*r*=–0.157; *P*=.01), social support (*r*=0.138; *P*=.03), and social media exposure duration (*r*=–0.174; *P*=.005). The univariate analyses between covariates and each score of self-management dimensions are presented in Table S5 in Multimedia Appendix 1.

**Associations Between Time Exposure and Self-Management**

**Figure 1:**
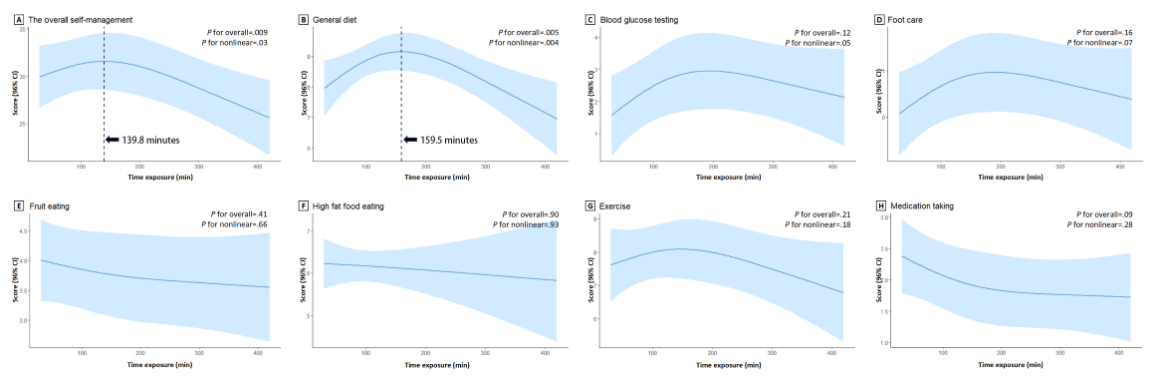
Associations between time exposure and the self-management dimensions. (A) The overall self-management adjustments include working status, diabetes duration, pain, medication related to diabetes, self-efficacy, perceived stress, social support, and social media exposure duration. (B) General diet adjustments include marital status, health-related content exposure to traditional media, and self-efficacy. (C) Blood glucose testing adjustments include diabetes duration, medication related to diabetes, and self-efficacy. (D) Foot care adjustments include diabetes duration, medication related to diabetes, complications of diabetes, and self-efficacy. (E) Fruit-eating adjustments include educational background, working status, monthly household income per capita, exposure to multiplatforms, and social support. (F) High-fat food eating adjustments include daily smartphone use time and traditional media exposure. (G) Exercise adjustments include sex, pain, complications of diabetes, self-efficacy, social support, perceived stress, and social media exposure duration. (H) Medication-taking adjustments include sex, diabetes duration, medication related to diabetes, and complications of diabetes.

As shown in Figure 1, time exposure and the sum score of self-management followed an approximate L-shaped dextrorotatory trend (*P* for nonlinear=.03). The trend started with a marginal and almost linear increase between 10 minutes and 139.8 minutes, followed by a sharp and almost linear decline until 600 minutes. The sample was then divided into the ≤139.8 minutes group and the >139.8 minutes group for further analyses. The nonlinear relationship between time exposure and the general diet score was statistically significant (*P* for nonlinear=.004).

### Associations Between Diabetes-Related Content Exposure and Self-Management

One participant who fell into the category of harmful content exposure was not representative and was therefore excluded from the subsequent analyses. In the full sample, compared with irrelevant content exposure, hyperbeneficial content exposure was associated with the higher sum score of self-management (B=3.46, 95% CI 0.71 to 6.21); while the association between hypobeneficial content exposure and the sum score of self-management was positive but not statistically significant (B=0.17, 95% CI –3.54 to 3.88; Table 3). In subdimensional analyses, hyperbeneficial content exposure was associated with the higher score of general diet (B=1.51, 95% CI 0.54 to 2.49) and blood glucose testing (B=1.31, 95% CI 0.25 to 2.38). The association between hyperbeneficial content exposure and the foot care score was not statistically significant (B=0.75, 95% CI –0.10 to 1.52; Table S6 in Multimedia Appendix 1).

**Table 3:** Associations between diabetes-related content exposure and the sum score of self-management^a^.

Diabetes-related content exposure (reference irrelevant)			B^b^ (SE; 95% CI)		*P* value
**Full sample (N=256^c^),**					
	Hypobeneficial	0.49 (1.91; –3.26 to 4.25)		.80	
	Hyperbeneficial	3.46 (1.40; 0.71 to 6.21)		.01^d^	
**The ≤139.8 min group (n=147)**					
	Hypobeneficial	–0.62 (2.26; –5.09 to 3.85)		.77	
	Hyperbeneficial	2.22 (1.73; –1.20 to 5.64)		.18	
**The >139.8 min group (n=109)**					
	Hypobeneficial	5.38 (3.76; –2.10 to 12.85)		.16	
	Hyperbeneficial	7.27 (2.89; 1.54 to 13.00)		.01^d^	

^a^Covariates adjusted include working status, diabetes duration, pain, medication related to diabetes, self-efficacy, perceived stress, social support, and social media exposure duration.

^b^B: unstandardized regression coefficient.

^c^One case with harmful exposure was excluded.

^d^*P* values less than the Bonferroni-corrected level of significance of <.017.

In subgroup analyses, among participants with excessive time spent on social media, compared with irrelevant content exposure, the association between hyperbeneficial content exposure and the sum score of self-management remained robust (B=7.27, 95% CI 1.54-13.00; Table 3); while hyperbeneficial content exposure was not significantly associated with the score of general diet at the Bonferroni-adjusted level (B=2.38, 95% CI 0.36-4.40; Table S7 in Multimedia Appendix 1).

## Discussion

### Principal Findings

In this study, we reported that time exposure and content exposure on social media were associated with self-management in older adults with T2DM. Specifically, spending less than 139.8 minutes on social media per day was beneficial for self-management, but beyond that, increased time exposure would decrease self-management scores. Exposure to hyperbeneficial content was positively associated with overall self-management in both the full sample and among those using social media for more than 139.8 minutes per day.

Previous studies relied solely on subjective assessments [32,33]. It is often assumed that older adults are more sensitive to time, yet this assumption is not empirically confirmed. In this study, we did not take this assumption as a given and instead assessed both objective and subjective time simultaneously. Through comparison, we found these 2 measures to be highly correlated. In addition, considering that subjective time offers greater practical feasibility in community settings and better comparability with existing literature, we chose to use subjective time throughout our analyses. By identifying the strong associations between these 2 variables, we determined to use subjective time exposure in this study because it is feasible to assess. Instead of following the traditional paradigm, that is, assuming the linear relationships between time exposure and health behaviors [34], we used the restricted cubic spline and found an approximate L-shaped dextrorotatory trend between time exposure and the overall self-management. This suggests that excessive exposure to social media may compromise the self-management of adults with T2DM. We also found that excessive time exposure would significantly associate with a decline in participants’ adherence to a diabetes diet. Understanding the mechanism underlying these findings falls outside the scope of this study, but some information in the literature would facilitate unraveling the hidden story in the black box. For example, excessive exposure to social media would reflect individuals’ suboptimal executive function [35], and the latter would result in unsatisfactory diabetes self-management [36]. As for the general diet, social media is a popular platform for unhealthy food advertising, and excessive exposure might increase the likelihood of individuals becoming its audience and then being driven away from healthy diets [37,38]. Future studies may corroborate these findings and advance science by adding robust evidence on the mechanisms between time spent on social media and self-management of older adults with T2DM.

By sorting out content exposure gathered through a semiclosed question and records documented on social media, we found that 61.1% (157/257) had hyperbeneficial content exposure, including information about diabetes diet, complications, and treatment. With the high-resolution data, we found that compared with exposure to irrelevant content, participants exposed to hyperbeneficial content exhibited significantly better performance on overall self-management and subdimensions of general diet and blood glucose testing; the positive effects of hyperbeneficial content exposure on self-management remained robust among those with excessive time exposure. Cultivation theory posits that media exposure would reshape individuals’ awareness and behaviors through information dissemination [13]. Consistent with this proposition, we found that exposure to hyperbeneficial content was significantly associated with better overall self-management, which validates the role of social media in shaping health behaviors. Furthermore, this study extends the proposition of cultivation theory by revealing the nonsignificant effects of hypobeneficial content and the negative effect of prolonged time exposure. If high-quality cohort studies or interventions confirm the findings from our study, this evidence may support media prescription in community settings. For example, health care providers may implement content dissemination programs through partnerships with social media platforms or curated content libraries for patients. It may also help social media servers optimize user profile capture, for example, by expanding information collection to include illness information and enhancing the recommender systems.

Subdimensional analyses revealed a positive trend between hyperbeneficial content exposure and foot care outcome, although this result was not statistically significant (*P*=.05). Foot care is often regarded as one of the most challenging practices encountered by health care providers [39,40], and this emerging trend nonetheless merits further investigation. Our results showed that only 2% (5/257) of the participants reported exposure to foot care content on social media. Empirical evidence showed that foot care information on social media was limited, but the available content was useful and highly reliable [41]. We failed to capture the association between hyperbeneficial content exposure and the practice of foot care, but this may be attributed to the small sample of individuals with foot care information exposure in our study. Gaps between diabetic foot care and other dimensions of self-management should be addressed, and we hope that health care providers may take their roles in posting high-quality foot care information on social media, getting familiar with and updating the information, and recommending it to older adults with T2DM.

### Limitations

Although the findings of this study are strengthened by addressing the methodological pitfalls and content gaps in the literature, some limitations should be considered. First, several novel hypotheses were proposed regarding the relationships between social media exposure and self-management among older adults with T2DM. However, this study is cross-sectional, and future longitudinal or experimental research is required to determine the causality of these relationships. Second, most of the adults in our study had higher educational backgrounds, better economic status, and lived in urban areas. While individuals with these characteristics are the primary users of social media [42], this demographic skew may limit the extrapolation of our findings to populations with different characteristics, such as those living in rural areas and individuals with lower socioeconomic status. Third, we operationalized social media exposure as time exposure and content exposure, as often assessed in the literature. However, social media exposure may include other parameters, such as online social engagement, which reflects the *social* feature of social media exposure. Future research could measure variables such as chatting frequency on chatting, posting, and gaming, and investigate their influences on older adults’ self-management. A concept analysis of social media exposure should be conducted to lay the methodological foundation for research in this area.

### Conclusions

This study reveals the dual effects of social media exposure on the self-management of older adults with T2DM. Self-management was aggravated when daily social media use time exceeded 139.8 minutes. However, exposure to hyperbeneficial content facilitated self-management, even under excessive time exposure. These findings provide new evidence suggesting that social media has the potential to serve as a valuable source for enhancing the self-management of older adults with T2DM. Further high-quality cohort studies are needed to confirm these findings, which may contribute to the development of strategies to guide appropriate social media exposure in older adults with T2DM to enhance their self-management.

## Appendix

Multimedia Appendix 1 Supplemental tables and figures.

## Abbreviations

STROBE: Strengthening the Reporting of Observational Studies in Epidemiology
T2DM: type 2 diabetes mellitus
