# Supplementary material for: The Dual Impact of Time and Content Exposure of Social Media on Diabetes Self-Management in Older Adults: Cross-Sectional Study
Source: JMIR Aging. 2025 Sep 18;8:e67312. doi: 10.2196/67312 (PMC12491892; doi:10.2196/67312)
Supplement: Multimedia Appendix 1 [file aging_v8i1e67312_app1.docx]

**Supplemental tables and figures**

[Figure S1. Process of Q-methodology.](#_Toc203919266)

[Table S1. Interpretations on social media exposure information.](#_Toc203919267)

[Table S2. Coding and grading on sample characteristics.](#_Toc203919268)

[Table S3. Summary of assessment tools used on assessing psychological variables.](#_Toc203919269)

[Table S4. The classification and relevant scores of contents.](#_Toc203919270)

[Table S5. Factors associated with scores of self-management dimensions.](#_Toc203919271)

[Table S6. Associations between diabetes-related content exposure and scores of the self-management dimension in 256 older adults with type 2 diabetes mellitus.](#_Toc203919272)

[Table S7. Associations between diabetes-related content exposure and the score of general diet.](#_Toc203919273)

[Reference](#_Toc203919274)


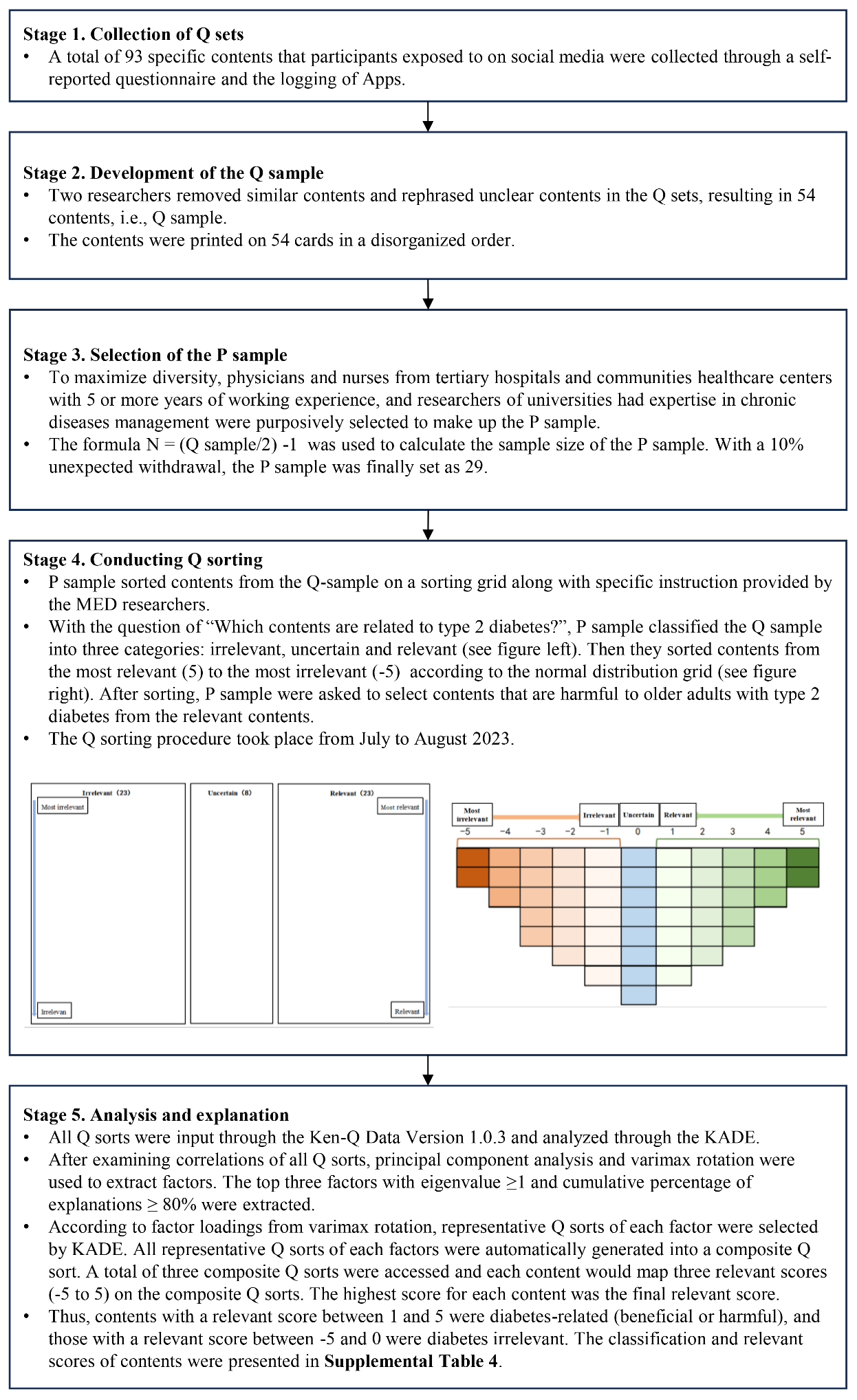
Figure S1. Process of Q-methodology.

Table S1. Interpretations on social media exposure information.

| Variable | Interpretation | Question(s) |
| --- | --- | --- |
| Social media exposure duration (years) | A continual variable to measure years from the first-time participants signed up for a social media account to now. | The variable was assessed by checking the register information kept on short-form video Apps, and the earliest register time was taken into account. |
| Exposure to multi-platforms | A categorial variable to evaluate whether a participant uses more than one social media Apps. (0 = No, 1 = Yes) | The variable was assessed by a semi-opened question “Please choose the short-form video Apps you used” with alternative options and a blank space for the respondent writing their own answer. |
| The feature of exposure | A categorial variable to evaluate the type of social media participants exposed to. (0 = Video-based, 1 = Video- and Text-based) | Judged based on descriptions of short-form video Apps. |
| Subjective exposure time (minutes) | A continuous variable to measure the average time participants spent on short-form video Apps per day. | The variable was assessed by a self-reported question “How long do you spend on short-form video Apps per day on average?” |
| Objective exposure time (minutes) | A continuous variable to measure the average time recorded on short-form video Apps per day. | The variable was obtained by dividing the one-week exposure time documented by the social media Apps by 7. |
| Diabetes-related content exposure | A categorical variable to evaluate the exposure of diabetes-related content on short-form video Apps. | General information about participants’ content exposure was collected through a semi-opened question “Please choose all the contents you watched on short-form video Apps” with 47 pre-prepared options and space for additional comments. In addition, participants' following, liking, bookmarking and browsing history on short-form video Apps were gathered to supplement their self-reported data. Q-methodology was then used to create a categorical variable named diabetes-related content exposure. |

Table S2. Coding and grading on sample characteristics.

| Characteristics | Coding |
| --- | --- |
| **Social demographic variables** | |
| Sex | 0 = Male, 1 = Female |
| Age (years) | Continuous |
| Educational background | 1 = Elementary school or below, 2 = Middle school, 3 = High school, 4 = College or above |
| Living status | 0 = Living alone, 1 = Living with family, relatives or friends |
| Marital status | 0 = Married, 1 = Unmarried/Divorced/Widowed |
| Working status | 0 = Retired, 1 = Still working |
| Monthly household income per capita (Chinese Yuan) | 1 = <2000, 2 = 2000-4000, 3 = >4000 |
| **Health-related variables** | |
| BMI | 1 = <24.0, 2 = 24.0–28.0, 3 = ≥28.0 |
| Pain | 0 = No, 1 = Yes |
| Diabetes duration (years) | Continuous |
| Medication related to diabetes | 1 = Insulin injections, 2 = Oral medicine, 3 = None |
| Complications of diabetes | 0 = No, 1 = Yes |
| Multimorbidity | 0 = No, 1 = Yes |
| Smoking history | 1 = Be smoking, 2 = Quit smoking, 3 = Non-smoker |
| Sleep duration (hours) | Continuous |
| **Traditional media usage** | |
| Traditional media exposure | 0 = No, 1 = Yes |
| Health-related content exposure on traditional media | 0 = No, 1 = Yes |
| **Daily smartphone use time (minutes)** | Continuous |

Table S3. Summary of assessment tools used on assessing psychological variables.

| Variable | Assessment tool | Scaling format | Number of items | Interpretation |
| --- | --- | --- | --- | --- |
| Self-efficacy | General Self-Efficacy Scale [1,2] | A 4-point scale, ranging from 1 (not at all true) to 4 (exactly true) | 10 | The sum score ranged between 10 and 40, with a higher score indicating a better self-efficacy. |
| Perceived stress | Perceived Stress Scale [3] | A 5-point scale, ranging from 0 (never) to 4 (always). | 14 | The sum score ranges between 0 and 56, with a higher score indicating a higher perceived stress. |
| Social support | Social Support Rating Scale [4] | Items 1 to 4 and items 8 to 10 are graded from 1 to 4 based on the option selected. Item 5 is divided into five subitems, each grading from 1 = none to 4 = full support. Item 6 and item 7 are multiple-choice questions; each item is graded from 0 to 9. | 10 | The sum score ranges between 12 and 66, with a higher score indicating a more social support. |

Table S4. The classification and relevant scores of contents.

| **Diabetes-related content** | | | | | | **Diabetes-irrelevant content** | | |
| --- | --- | --- | --- | --- | --- | --- | --- | --- |
| Beneficial content | Relevant score | Exposure rate (%) | Harmful content | Relevant score | Exposure rate (%) | Irrelevant content | Relevant score | Exposure rate (%) |
| **Hyperbeneficial contents** | | | Cooking instructions on food high in oil, salt and added sugar | 2 | 3.9% | Information on gastroenterology (spleen and stomach disorders, or constipation) | 0 | 0.8% |
| Information on diabetes diet | 5 | 49.4% |  |  |  |  |  |  |
| Information on diabetes complications | 5 | 5.8% | Misleading drug advertising | 2 | 3.5% | Methods of post-COVID recovery | 0 | 3.9% |
| Diabetes treatment | 5 | 46.3% | Recommendation of liquor and beer | 1 | 0.4% | Information on urology | 0 | 0.8% |
| Exercise guidance for diabetes | 4 | 38.5% |  | | | Information on oncology | 0 | 0.8% |
| Information on blood-glucose testing | 4 | 9.7% |  |  |  | Stories about family and friendship | 0 | 1.6% |
| Information on diabetes symptoms | 4 | 0.4% |  |  |  | Information on cardiac surgery | 0 | 1.6% |
| Foot ulcer care | 3 | 1.9% |  |  |  | Pain management | 0 | 0.4% |
| Advancements in diabetes treatment | 3 | 0.4% |  |  |  | Insomnia therapy | 0 | 1.6% |
| Methods of diabetes medication administration | 3 | 35.8% |  |  |  | Instruction on homestyle cooking | 0 | 21.4% |
| Information on side effects of diabetes medications | 3 | 3.1% |  |  |  | Guidance for folk exercises, i.e., finger exercises and emotional freedom techniques (tapping) | 0 | 9.3% |
| **Hypobeneficial content** | | |  |  |  | Information on dermatology | 0 | 0.8% |
| Case studies highlighting diabetes risks | 2 | 1.6% |  |  |  |  |  |  |
| Experiences shared by people with diabetes | 2 | 0.8% |  |  |  | The philosophy of life and death | 0 | 0.8% |
| Hypertension management | 2 | 25.7% |  |  |  | Shopping advertising | 0 | 1.6% |
| Hyperlipidemia management | 2 | 7.4% |  |  |  | Management of Chronic Obstructive Pulmonary Disease | -1 | 0.8% |
| Cerebrovascular disease management | 2 | 18.7% |  |  |  | Arthritis management | -1 | 1.2% |
| Chronic kidney disease management | 2 | 0.8% |  |  |  | Asthma management | -1 | 0.8% |
| Cooking instruction on light diets | 2 | 28.8% |  |  |  | Disease treatment folk remedies  (*continued)*  (*continued)* | -1  (*continued)* | 0.8% |
| Workout guide on brisk walking, square dancing and Tai Chi, workout plan for weight loss, and sports events | 1 | 46.3% |  |  |  | Information on orthopedics and spine surgery | -1 | 1.2% |
| Information on health insurance policies of diabetes | 1 | 1.2% |  |  |  | Mental health counseling | -1 | 0.4% |
| Coronary heart disease management | 1 | 1.9% |  |  |  | Friends posts on social media | -1 | 26.1% |
| Traditional Chinese medicine practice (e.g., diet therapy, acupuncture and moxibustion, or Tui na massage) | 1 | 28.4% |  |  |  | Leisure activities sharing including traveling, fishing, playing chess, taking photos, and flower arranging | -1 | 10.5% |
| Comprehensive health management | 1 | 3.1% |  |  |  | Information on Parkinson | -2 | 0.4% |
| Information on endocrine diseases | 1 | 1.6% |  |  |  | Household tips and common knowledge | -2 | 9.4% |
| Inspirational and positive energy stories | 1 | 1.6% |  |  |  | Game commentaries | -2 | 1.2% |
|  |  |  |  |  |  | Elderly care policies | -2 | 0.4% |
|  |  |  |  |  |  | Business strategy | -3 | 0.8% |
|  |  |  |  |  |  | Information on skincare and apparel | -4 | 10.2% |

Table S5. Factors associated with scores of self-management dimensions.

| Characteristics | Group | Dimensions of self-management | | | | | | | | | | | | | |
| --- | --- | --- | --- | --- | --- | --- | --- | --- | --- | --- | --- | --- | --- | --- | --- |
|  |  | General diet | | Fruit eating | | High fat food eating | | Exercise | | Blood-glucose testing | | Foot care | | Medication taking | |
|  |  | *r/Z/H* | *P* | *r/Z/H* | *P* | *r/Z/H* | *P* | *r/Z/H* | *P* | *r/Z/H* | *P* | *r/Z/H* | *P* | *r/Z/H* | *P* |
| Sex | Male  Female | 0.222 | .82 | 1.217 | .22 | -1.316 | .19 | -2.849 | .004 | -0.116 | .91 | -0.737 | .46 | -2.951 | .003 |
| Age, y |  | 0.093 | .14 | -0.058 | .36 | -0.058 | .35 | -0.101 | .11 | -0.031 | .62 | -0.084 | .18 | 0.026 | .68 |
| Educational background | Elementary school or below  Middle school  High school  College or above | 1.219 | .75 | 7.939 | .05 | 3.279 | .35 | 2.531 | .47 | 4.904 | .18 | 3.019 | .39 | 2.468 | .48 |
| Living status | Living alone  Living with family, relatives or friends | -0.962 | .34 | -0.678 | .49 | -1.205 | .23 | 0.630 | .53 | -0.184 | .85 | 0.259 | .80 | -0.334 | .74 |
| Marital Status | Married  Unmarried/Divorced/Widowed | 2.397 | .02 | -0.433 | .67 | 0.325 | .75 | -0.844 | .40 | 0.682 | .50 | -1.093 | .28 | -0.631 | .53 |
| Working status | Retired  Still working | 1.100 | 0.27 | 2.023 | .04 | -0.401 | .69 | 1.436 | .15 | 1.350 | .18 | 0.912 | .36 | 1.874 | .06 |
| Monthly household income per capita, CNY | ＜2000  2000-4000  ＞4000 | 1.349 | 0.51 | 6.398 | .04 | 5.302 | .07 | 4.858 | .09 | 1.905 | .39 | 2.356 | .31 | 2.758 | .25 |
| BMI | <24.0  24.0–28.0  ≥ 28.0 | 4.378 | .11 | 0.204 | .90 | 5.628 | .06 | 9.325 | .009 | 3.918 | .14 | 2.001 | .37 | 1.370 | .50 |
| Pain | No  Yes | -0.511 | .61 | -1.492 | .14 | -0.574 | .57 | -5.022 | <.001 | -1.174 | .24 | -1.023 | .31 | -1.568 | .12 |
| Diabetes duration, y |  | 0.095 | .13 | -0.004 | .95 | -0.013 | .83 | 0.003 | .96 | 0.277 | <.001 | 0.152 | .02 | 0.438 | <.001 |
| Medication related to diabetes | None  Oral medicine  Insulin | 3.696 | .16 | 0.684 | .71 | 0.203 | .90 | 2.600 | .27 | 18.773 | <.001 | 10.652 | .005 | 122.263 | <.001 |
| Complications of diabetes | No  Yes | 0.349 | .73 | -1.015 | .31 | -0.463 | .64 | -1.983 | .05 | -0.039 | .97 | 2.183 | .03 | 1.991 | .05 |
| Multimorbidity | No  Yes | 0.673 | .50 | 1.062 | .29 | 0.038 | .97 | -1.220 | .22 | -1.197 | .84 | -0.432 | .67 | -0.269 | .79 |
| Smoking history | Be smoking  Quit smoking  Non-smoker | 4.339 | .11 | 2.748 | .25 | 1.494 | .47 | 1.883 | .39 | 0.353 | .84 | 5.048 | .08 | 0.757 | .69 |
| Sleep duration, h |  | -0.017 | .79 | 0.076 | .22 | 0.047 | .46 | 0.059 | .34 | -0.012 | .85 | -0.086 | .17 | 0.011 | .86 |
| Traditional media exposure | No  Yes | -1.879 | .06 | 1.829 | .07 | 2.391 | .02 | -0.509 | .61 | -0.695 | .49 | 0.190 | .85 | 0.941 | .35 |
| Health-related content exposure on traditional media | No  Yes | 2.713 | .007 | 1.933 | .05 | -1.052 | .29 | 0.814 | .42 | -0.787 | .43 | -0.675 | .50 | 0.434 | .66 |
| Daily smartphone time use, min |  | -0.016 | .80 | 0.058 | .36 | 0.245 | <.001 | 0.028 | .65 | 0.102 | .10 | 0.077 | .22 | -0.087 | .16 |
| Social media exposure duration, y |  | 0.106 | .09 | 0.080 | .20 | -0.020 | .76 | 0.124 | .05 | 0.105 | .09 | 0.099 | .11 | 0.054 | .39 |
| Exposure to multi-platforms | No  Yes | -1.189 | .23 | 2.250 | .02 | 1.152 | .25 | 0.243 | .81 | 0.595 | .55 | 0.893 | .37 | -0.394 | .69 |
| The feature of exposure | Video-based  Video- and Text-based | -0.663 | .51 | -0.369 | .71 | 1.764 | .08 | 1.427 | .15 | 0.935 | .35 | 0.615 | .54 | -1.404 | .16 |
| Self-efficacy |  | 0.184 | .003 | 0.075 | .23 | -0.073 | .24 | 0.245 | <.001 | 0.144 | .02 | 0.129 | .04 | 0.064 | .30 |
| Perceived stress |  | -0.072 | .25 | -0.132 | .03 | -0.020 | .75 | -0.283 | <.001 | -0.084 | .18 | -0.057 | .36 | -0.034 | .59 |
| Social support |  | 0.069 | .27 | 0.175 | .005 | 0.007 | .91 | 0.167 | .007 | 0.042 | .51 | -0.019 | .77 | -0.005 | .93 |

Table S6. Associations between diabetes-related content exposure and scores of the self-management dimension in 256 older adults with type 2 diabetes mellitus.

| Diabetes-related content exposure  (ref. irrelevant) | General diet^a^ | | Blood-glucose testing^b^ | | Foot care^c^ | | Fruit eating^d^ | | High fat food eating^e^ | | Exercise^f^ | | Medication taking^g^ | |
| --- | --- | --- | --- | --- | --- | --- | --- | --- | --- | --- | --- | --- | --- | --- |
|  | B (95% CI) | *P* | B (95% CI) | *P* | B (95% CI) | *P* | B (95% CI) | *P* | B (95% CI) | *P* | B (95% CI) | *P* | B (95% CI) | *P* |
| Hypobeneficial | 0.14 (-1.20 to 1.49) | .84 | -0.15 (-1.63 to 1.33) | .84 | -0.15 (-1.21 to 0.91) | .17 | 0.12 (-0.51 to 0.76) | .71 | -0.19 (-0.81 to 0.43) | .55 | 0.91 (-0.51 to 2.33) | .21 | -0.02 (-0.65 to 0.64) | .99 |
| Hyperbeneficial | 1.51 (0.54 to 2.49) | *.002*^h^ | 1.31 (0.25 to 2.38) | *.016*^h^ | 0.75 (-0.10 to 1.52) | .05 | 0.22 (-0.26 to 0.69) | .38 | 0.13 (-0.31 to 0.58) | .56 | 1.04 (0.02 to 2.07) | .05 | -0.14 (-0.60 to 0.32) | .55 |

^a^General diet adjustments include marital status, health-related content exposure on traditional media and self-efficacy.

^b^Blood-glucose testing adjustments include diabetes duration, medication related to diabetes and self-efficacy.

^c^Foot care adjustments include diabetes duration, medication related to diabetes, complications of diabetes and self-efficacy.

^d^Fruit eating adjustments include educational background, working status, per capita monthly income, exposure to multi-platforms and social support.

^e^High fat food eating adjustments include daily smartphone use time and traditional media exposure.

^f^Exercise adjustments include sex, pain, complications of diabetes, self-efficacy, social support, perceived stress, and social media exposure duration.

^g^Medication taking adjustments include sex, diabetes duration, medication related to diabetes, and complications of diabetes. One case with harmful exposure were excluded.

Italics font indicates *P* values less than the Bonferroni-corrected level of significance of <.017.

^h^P values less than the Bonferroni-corrected level of significance of <.017.

Table S7. Associations between diabetes-related content exposure and the score of general diet (N=256^a^).

|  | Diabetes-related content exposure  (ref. irrelevant) | B | SE | 95%CI | *P* |
| --- | --- | --- | --- | --- | --- |
| General diet^b^ |  |  |  |  |  |
| The ≤159.4 minutes group (*n =* 150) | Hypobeneficial  Hyperbeneficial | 0.24  1.51 | 0.81  0.58 | (-1.35 to 1.83)  (0.37 to 2.65) | .77  *.009*^c^ |
| The >159.4 minutes group (*n =* 106) | Hypobeneficial  Hyperbeneficial | 0.45  2.38 | 1.34  1.03 | (-2.18 to 3.09)  (0.36 to 4.40) | .74  .02 |

^a^One case with harmful exposure were excluded.

^b^General diet adjustments include marital status, health-related content exposure on traditional media and self-efficacy.

^c^P values less than the Bonferroni-corrected level of significance of <.017.

Reference

1. Zhang JX, Schwarzer R. Measuring optimistic self-beliefs: A Chinese adaptation of the General Self-Efficacy Scale. Psychol Int J Psychol Orient. Japan: Psychologia Society; 1995;38(3):174–181.

2. Ma M, Ai Z, Shi Z. Reliability and validity of General Self-Efficacy Scale (Chinese version) in middle-aged and elderly patients with type 2 diabetes. J Tongji Univ Sci. Sep 15, 2022;43(4):515–520. doi: 10.12289/j.issn.1008-0392.21411

3. Yang T, Huang H. An epidemiological study on stress among urban residents in social transition period. Chin J Epidemiol. Sep 2003;24(9):760–764. PMID:14521764

4. Xiao S. Theoretical basis and research applications of the Social Support Rating Scale (SSRS). J Clin Psychiatry. 1994;4(2):98–100.
